# Indirect Additive Manufacturing: A Valid Approach to Modulate Sorption/Release Profile of Molecules from Chitosan Hydrogels

**DOI:** 10.3390/polym14132530

**Published:** 2022-06-21

**Authors:** Mariana F. Moreira, Akel F. Kanaan, Ana P. Piedade

**Affiliations:** CEMMPRE-Department of Mechanical Engineering, University of Coimbra, 3030-788 Coimbra, Portugal; mariana.fmoreira98@gmail.com (M.F.M.); ana.piedade@dem.uc.pt (A.P.P.)

**Keywords:** hierarchical surface roughness, chitosan hydrogel, indirect additive manufacturing, dye removal, drug delivery

## Abstract

This work studied the influence of hydrogel’s physical properties (geometry and hierarchical roughness) on the in vitro sorption/release profiles of molecules. To achieve this goal, chitosan (CS) solutions were cast in 3D-printed (3DP) molds presenting intricate shapes (cubic and half-spherical with/without macro surface roughness) and further immersed in alkaline solutions of NaOH and NaCl. The resulting physically crosslinked hydrogels were mechanically stable in aqueous environments and successfully presented the shapes and geometries imparted by the 3DP molds. Sorption and release profiles were evaluated using methyl orange (MO) and paracetamol (PMOL) as model molecules, respectively. Results revealed that distinct MO sorption/PMOL release profiles were obtained according to the sample’s shape and presence/absence of hierarchical roughness. MO sorption capacity of CS samples presented both dependencies of hierarchical surface and geometry parameters. Hence, cubic samples without a hierarchical surface presented the highest (up to 1.2 × greater) dye removal capacity. Moreover, PMOL release measurements were more dependent on the surface area of hydrogels, where semi-spherical samples with hierarchical roughness presented the fastest (~1.13 × faster) drug delivery profiles. This work demonstrates that indirect 3DP (via fused filament fabrication (FFF) technology) could be a simple strategy to obtain hydrogels with distinct sorption/release profiles.

## 1. Introduction

Along with the Internet of Things, artificial intelligence, and robotics, additive manufacturing (AM) is a disruptive technology that is a pillar of the fourth industrial revolution (Industry 4.0) [1]. AM technologies, also commonly known as 3D printing, allow the fabrication of tailor-made complex structures with intricate shapes and geometries, with a high degree of automation and reproducibility, in a short time, and with low waste production and efficient reuse of recycled material [2]. A three-dimensional object is obtained via layer-by-layer material deposition from a 3D computer-aided design (CAD) file. Of the seven AM processes, material extrusion is the most known, mainly due to the worldwide spread of the specific technology of fused filament fabrication (FFF). However, all 3D printing technologies have been explored for use in a number of applications, namely fashion [3], food industry [4], medicine [5], automotive, and aerospace sectors [6].

Among the aforementioned AM applications, the utilization of 3DP systems for drug delivery [7,8] and wastewater treatment [9,10] have been receiving significant attention. In these cases, the material jetting process and FFF technology are the most used in manufacturing these polymeric-based systems due to their affordability, availability, and ease of use. The conjugation of 3DP technology with wastewater and drug delivery systems allows the design of complex polymer-based structures in which the physical cues can induce functionality that in the last century could only achieved by chemical modification with hazardous reagents and radiation [11,12,13]. These finely tuned geometries and topographies make it possible to customize physical properties and prompt adjustable sorption/diffusion profiles due to differences in surface area, surface roughness, and volume. Different synthetic and natural polymers have been studied for the design of advanced 3DP sorption and release systems, including poly(lactic acid) (PLA) [14,15,16], poly(vinyl alcohol) (PVA) [17,18,19,20,21], alginate [22], cellulose [23], and chitosan [24,25,26,27,28,29,30].

Due to its unique characteristics (biodegradability, biocompatibility, low toxicity, polycationic nature, and low cost) and versatility (broad processability, availability, and gel-forming capacity), chitosan (CS) has been extensively explored for biomedical and environmental applications such as drug delivery [31,32,33,34] and wastewater treatment systems [35,36]. Despite its advantages, CS possesses a pKa of ~6.5, which makes it soluble only under acidic conditions (pH_medium_ < pKa) and presents poor mechanical properties. CS is obtained from the deacetylation of chitin, the second most abundant natural polymer after cellulose, commonly found in the exoskeletons of arthropods and cell walls in fungi. It consists of a linear polysaccharide structure composed of randomly distributed β-(1-4)-linked D-glucosamine and N-acetyl-D-glucosamine glycosidic bonds. More recently, CS has been utilized in conjugation with 3DP technology in order to develop personalized systems regarding metallic ions removal (copper, lead, and mercury) [28,37] and protein/drug delivery (bovine serum albumin (BSA), ibuprofen, lidocaine hydrochloride, and diclofenac) [24,25,26,27] applications. These 3DP systems have a design advantage by presenting complex and intricate structures/shapes that influence the final material’s physicochemical and surface properties (swelling ratio, hierarchical roughness). Consequently, these influences modulate the adsorptive, diffusive, and erosive behavior of the developed platforms by improving the surface area accessibility.

Generally, sorption/release systems obtained via FFF technology are 3D printed in a direct approach [14,17,18,19,20,25]. In this case, the intended system is obtained by directly depositing a given material into the final 3D form. As an alternative, this work evaluates the feasibility of an indirect FFF printing approach to obtain CS hydrogels with different topographies and geometries. The objective of this work is to evaluate the influence of hydrogel’s geometry (semi-spherical and cubical) and surface topography (with and without hierarchical roughness achieved through the variation of the deposited layer height [38] of the mold) on the removal and drug delivery capacity of model molecules such as methyl orange (MO) and paracetamol (PMOL), respectively. To address this goal, 3DP molds were developed via FFF comprising different shapes, geometries, and surface roughness. This simple yet poorly explored strategy is expected to impart the mold’s physical properties onto the cured hydrogels and consequently induce distinct sorption/release profiles. Thus, it allows the indirect obtention of build-oriented CS hydrogels and avoids eventual thermal-induced drug degradation during direct material extrusion when drug delivery systems are envisaged.

## 2. Materials and Methods

### 2.1. Materials

All the reagents were purchase in Ordivelas, Portugal at a supplier (JMGS) that represents several companies, namely: chitosan (CS, Mw 50–190 kDa, degree of acetylation ≥ 90%, Acros Organics), paracetamol (PMOL, 98%, Acros Organics), acetic acid (AAc, 99–100%, Chem-Lab), sodium hydroxide (NaOH, Eka-AkzoNobel), sodium chloride (NaCl, 99.5%, Panreac Química SAU) and methyl orange (MO, Panreac AppliChem ITW Reagents). Natural acrylonitrile-butadiene-styrene (ABS) filament (d = 1.75 mm) was purchased from DoWire^®^ Company (Corroios, Portugal). All materials were used without further modification.

### 2.2. 3D Printing of the Molds

Several custom-made molds were printed to support the development of hydrogels with different topographies and geometries. ABS molds were manufactured in an FFF-based FlashForge Creator 3 equipment, using the following printing parameters: nozzle and bed temperature of 240 °C and 110 °C, respectively; printing speed of 40 mm/s; linear infill pattern with 25% density and layer thickness of 0.4 mm.

The printed molds were developed with a fixed volume of 2.5 mL in each well (independent of geometry). Each mold geometry presented a different theoretical surface roughness, namely 663.8 mm^2^ (semi-spherical), 1250.2 mm^2^ (rough semi-spherical), 943.5 mm^2^ (cubical) and 1704.2 mm^2^ (rough cubical). These values were calculated according to SolidWorks^®^ software.

To ensure higher contact between the molds and avoid CS solution leakage after casting, each half of the molds was polished to ensure better contact between them.

### 2.3. Synthesis of CS Hydrogels with Different Geometries and Surface Roughness

The synthesis of CS hydrogels was based on previously reported methods with some modifications [39,40,41]. The procedure was carried as follows: CS (% *w*/*v*) was dissolved in 35 mL of AAc solution (1% *v*/*v*). Then, the CS solution was stirred (1000 rpm) for 24 h at room temperature (~23 °C) to obtain a homogeneous viscous solution. Subsequently, 2.5 mL of the acidic CS solution was poured into each well of the 3DP molds (previously stored at −20 °C for 24 h). The molds containing the CS solutions were frozen at −20 °C for at least 48 h.

To promote physical crosslinking of CS, a gelling solution was prepared as follows: NaOH (10% *w*/*v*) and NaCl (2.5 M) were dissolved in 300 mL of Milli-Q^®^ water. The electrolyte solution was stirred (800 rpm) at room temperature (~23 °C) for 24 h and then stored at 4 °C for at least 48 h.

Furthermore, the molds containing the frozen CS solutions (3% *w*/*v*) were immersed in the previously prepared gelling solution (300 mL) for 48 h at 4 °C. The resulting hydrogels were carefully removed from the molds and washed in distilled water (~1 L of freshwater replaced twice a day for 4 days) to remove the remaining NaOH/NaCl until neutralization was achieved. The pH of the washing solution was monitored with pH test strips every washing cycle.

Hydrogel samples were coded according to the pattern of each mold, namely semi-spherical (CS-Sph), semi-spherical with hierarchical roughness (CS-Sph_r_), cubical (CS-Cub), and cubical with hierarchical roughness (CS-Cub_r_).

### 2.4. Characterization of the Prepared CS Hydrogels

#### 2.4.1. Chemical Analysis

Attenuated total reflection Fourier transform infrared (FTIR-ATR) spectra were acquired using a spectrometer (Bruker, model Alpha II, Leipzig, Germany) at 24 scans and 4 cm^−1^ resolution between 400 and 4000 cm^−1^. The spectra obtained were analyzed using the software OPUS from Bruker.

#### 2.4.2. Swelling

The water sorption capacity (WSC) of the prepared hydrogels was measured at room temperature (~23 °C) in bi-distilled water. The previously washed hydrogels (at sorption equilibrium) were weighed (after removing the excess surface water with a filter paper) and dried at 60 °C for 48 h. Afterward, the dried samples were weighed and the *WSC* was calculated as follows (Equation (1)):

(1) $$WSC\;\left(\%\right)=\frac{W_{eq}-W_{d}}{W_{d}}\times 100$$

where *W_eq_* is the weight of the samples at equilibrium and *W_d_* is the weight of the samples after the drying process. Measurements were replicated twice, and the results are presented in g_water_/g_dried hydrogel_ (%).

#### 2.4.3. Sorption Capacity of the CS Hydrogels

The capacity of the synthesized polycationic hydrogels to adsorb/absorb anionic molecules was investigated using methyl orange (MO, Mw = 327.34 g/mol) as a model molecule. The hydrogels (at WSC equilibrium) were immersed in 15 mL of MO aqueous solution (0.0625 mM) and stored at room temperature (~23 °C). Measurements were carried out in a UV/VIS JASCO 530 spectrophotometer, Tokyo, Japan (at λ = 464 nm) and performed at predetermined time intervals. The amount of MO removed by the polycationic hydrogels from each solution was quantified using a previously obtained calibration curve of MO in bi-distilled water ((MO) = 0.5402 × Abs, R^2^ = 0.999). The experiments were performed in duplicate, and the results are expressed in terms of MO concentration.

#### 2.4.4. Release Behavior of the CS Hydrogels

The in vitro release capacity of synthesized hydrogels was evaluated using paracetamol (PMOL, 151.16 g/mol) as a model molecule. PMOL was considered due to the following criteria: (a) nonionic nature (to avoid electrostatic interactions with protonated CS); (b) availability; (c) affordability; (d) high water solubility; and (e) easy UV-VIS quantification. Before the experiments, the hydrogels were immersed in a PMOL aqueous solution (0.03125 mg/mL) for 120 h at room temperature (~23 °C) to ensure drug adsorption. Then, each drug-loaded sample was carefully placed in 15 mL of bi-distilled water at room temperature (~23 °C). At predetermined intervals, aliquots (3 mL) of the release medium were collected and returned to the flask after each measurement. Measurements were carried out in a UV/VIS JASCO 530 spectrophotometer (Tokyo, Japan) (at λ = 241 nm). The amount of PMOL released from the polycationic hydrogels was quantified using a previously obtained calibration curve of PMOL in bi-distilled water ((PMOL) = 0.0161 × Abs, R^2^ = 0.999). The results of the experiments are expressed as a percentage of released PMOL.

#### 2.4.5. Statistical Analysis

One-way ANOVA test was used for statistical analysis of the data obtained in this work. Statistical significance was considered for *p*-values < 0.05.

## 3. Results and Discussion

### 3.1. 3D Printing of Molds in ABS Comprising Different Geometries with/without Macro Hierarchical Roughness

**Figure 1:**
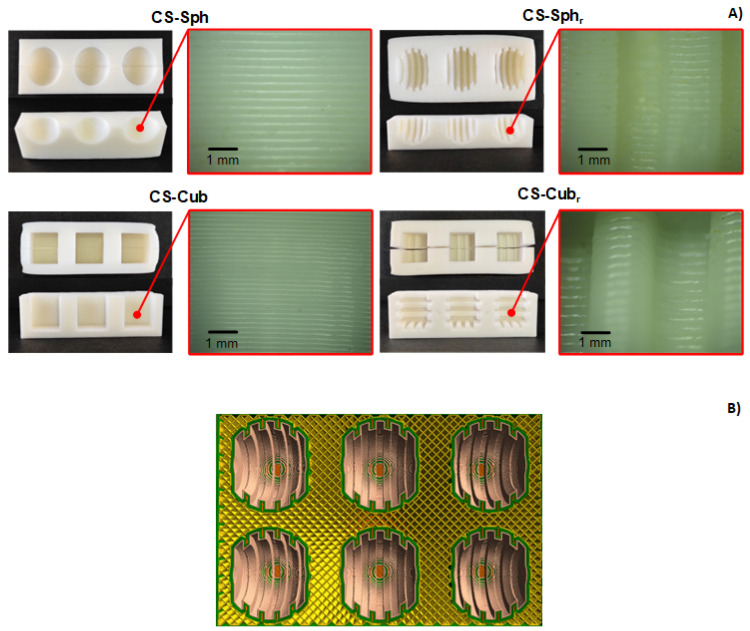
Top and cross-section views (left and right columns, respectively) of ABS 3DP molds featuring different shapes and geometries (**A**) and example of sliced perspective of 3DP mold (**B**).

Different molds with distinct geometries and surface topographies were successfully obtained by FFF 3DP (Figure 1A). It is possible to notice that molds (independently of geometry and shape) presented a rough surface due to the deposition of polymer layers during printing process (insert of Figure 1A). Therefore, it allowed the obtention of molds with macro hierarchical roughness, as in the case of Sph_r_ and Cub_r_ molds.

The molds did not present any evidence of structural degradation after immersion in strong alkaline solutions. This indicates that ABS present high chemical resistance (as expected) due to the acrylonitrile polymeric counterpart in its copolymeric structure [42].

### 3.2. Synthesis of CS Hydrogels with Different Geometries and Surface Roughness

**Figure 2:**
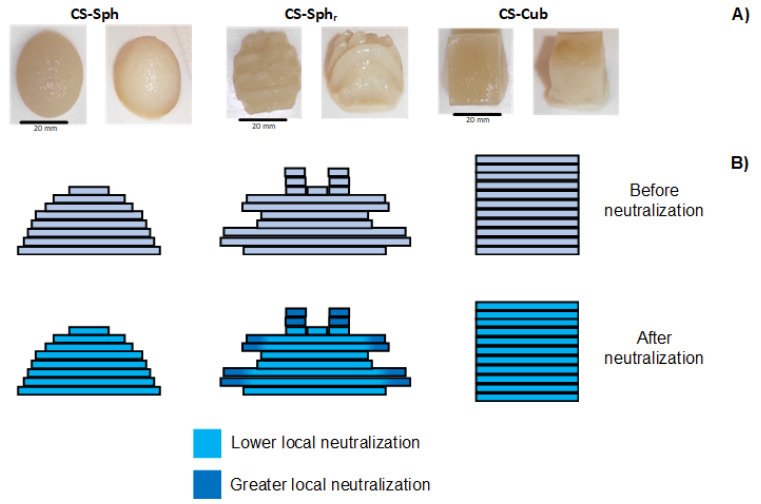
(**A**) Top and longitudinal view of the neutralized CS (3% *w*/*v*) hydrogels (with different shapes and geometries) represented in the left and right columns, respectively. (**B**) Suggested local neutralization gradient mechanism carried out during the neutralization step (NaOH 10% *w*/*v* + NaCl 2.5 M) of indirectly 3DP CS hydrogels.

After the synthesis procedure, the resulting CS hydrogels exhibited a soft and semi-solid structure. The samples showed high mechanical stability in bi-distilled water (for at least two months), which indicates that NaOH (10% *w*/*v*) + NaCl (2.5 M) solutions successfully induced physical crosslinking during the neutralization step (as expected). Nevertheless, the shape and geometry of the mold utilized during the crosslinking step influenced the surface integrity of the samples. From Figure 2A, it is noticeable that hydrogels prepared in semi-spherical and cubical molds without hierarchical surface roughness (CS-Sph and CS-Cub, respectively) presented a homogenous surface with well-defined geometries.

However, in the case of the hydrogels prepared in molds with hierarchical surface roughness, significant surface defects were observed especially in the case of CS-Cub_r_ samples (data not shown). These defects are directly related to the demolding step of the samples after the washing procedures. For this reason, CS-Cub_r_ samples were not considered in the present work.

### 3.3. Chemical Analysis

**Figure 3:**
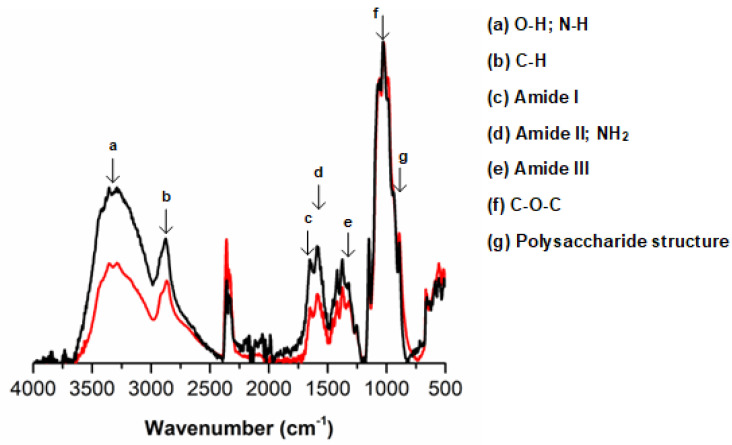
Normalized (at 1027 cm^−1^) FTIR-ATR spectra of NaOH neutralized CS (▬) and pristine CS (▬) samples.

To evaluate the influence of the neutralization step on the chemical composition of the hydrogels, FTIR-ATR analyses were conducted (Figure 3). In general, all samples presented the characteristic bands of chitosan [43,44], namely O-H stretch vibration overlapped with N-H elongation (3500–3300 cm^−1^), C-H elongation (2870 cm^−1^), amide I (C=O stretching at 1650 cm^−1^), amide II and amine (N-H bending at 1577 cm^−1^), amide III (C-N wagging at 1320 cm^−1^) [45], asymmetric CH_2_ and CH_3_ groups deformation (at 1419 and 1375 cm^−1^, respectively), asymmetric elongation of C-O-C glycosidic linkage (at 1027 cm^−1^) and polysaccharide structure (at 893 cm^−1^) [43,44,45,46,47,48]. When comparing both obtained spectra, it is evident that the neutralization step induced some chemical changes in the samples. Higher absorbance intensities were observed at the 3400–2900 and 1700–1200 cm^−1^ regions for neutralized CS samples compared to control (pristine CS). Regarding the first region, this variation is probably due to the higher presence of water in these samples (even after drying procedures), which indicates a high hydrophilic behavior, as will be discussed later. Concerning the second region, these peak intensity variations suggest an extra deacetylation process during the neutralization step in alkaline solution (NaOH 10% m/v + NaCl 2.5 M for 48 h) of the CS hydrogels. Thus, this additional deacetylation process would enhance the formation of anime groups (-NH_2_), resulting in higher peak intensities at ~1580 cm^−1^. To confirm this hypothesis, the degree of the acetylation (DA) of the samples was calculated from the absorbance ratio of bands at 1320 and 1420 cm^−1^ (A_1320_/A_1420_ × 100 = DA (%)) [49]. The neutralized samples presented a DA lower (~1.18 × lower) than the control which agrees with the results, indicating a higher concentration of anime groups in neutralized CS samples.

### 3.4. Swelling

The swelling behavior of the CS hydrogels (with a fixed volume of ~2.5 mL) was evaluated in bi-distilled water at room temperature (~23 °C). The samples (independent of the geometry and shape) presented a water uptake capacity of 1847.6 ± 40% after 7 days of measurements (at equilibrium). As expected, these results indicate a higher hydrophilic behavior of the prepared hydrogels. At aqueous media (pH ≈ 6.0) the protonation of amino groups (from NH_2_ to NH_3_^+^) of CS is prompted (pH_medium_ < pKa_CS_). As a result, electrostatic repulsion among CS polymer chains is attained, increasing the free volume. Thus, an osmotic pressure gradient occurs between the hydrogel and the aqueous media (greater inside the positively charged hydrogel) inducing a diffusion of water molecules towards the hydrogel to “dilute” charge density and attain equilibrium which ultimately, result in high water swelling capacity. The hydrogel swells until a certain threshold delimited by its mechanical elastic response and crosslinking degree [50].

The high water sorption capacity of the samples yields a soft and fragile structure. Consequently, the hydrogels with hierarchical roughness (CS-Sph_r_ and CS-Cub_r_) presented some surface defects after the demolding step, especially in the case of CS-Cub_r_ (as previously mentioned), due to the intricate structure of the molds. Finally, the samples presented mechanical stability with defined geometry and shape in aqueous media for at least one month at room temperature (~23 °C).

### 3.5. Sorption Capacity of the CS Hydrogels

**Figure 4:**
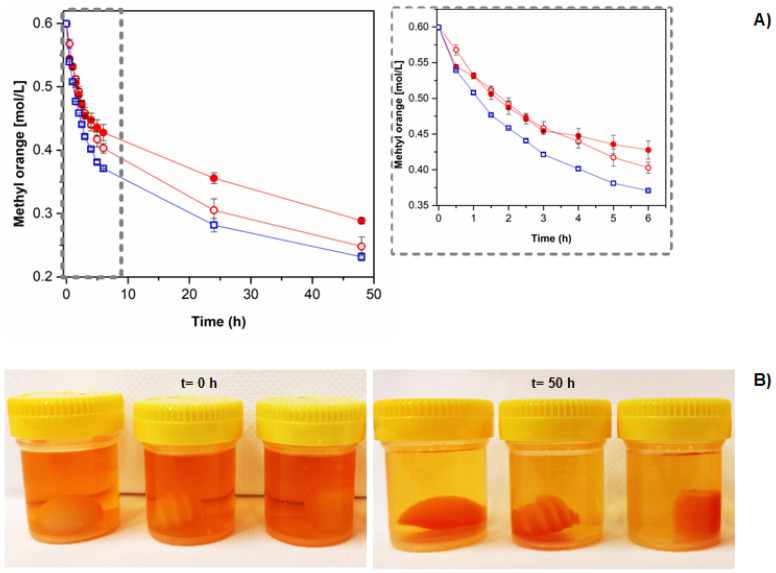
Effect of geometry and hierarchical roughness of polycationic hydrogels onto the sorption capacity of anionic model molecule (methyl orange [0.0625 mM]) at room temperature (~23 °C) (**A**) and illustration of each aqueous media before and after the contact with the samples (**B**). The hydrogels were coded as: CS-Sph (○), CS-Sph_r_ (●) and CS-Cub (☐).

The effect of the geometry and surface roughness on the sorption capacity of indirectly 3DP CS hydrogels was evaluated in vitro, and the results are presented in Figure 4. In general, all the samples presented dye removal capacity independent of the geometry and presence of the hierarchical rough surface. It indicates that the chemical nature of the hydrogels plays an important role in determining the adsorption capacity. The main mechanisms of anionic dye removal by the polycationic samples are diffusion (due to the concentration gradient of MO) and electrostatic interactions (between the anionic molecules of the dye with the positively charged amino groups of CS), which depends on the media’s pH. The pH of MO aqueous solutions was 5.5–6.0, which is below CS’s pKa of 6.5. In this case, the amine groups of CS are protonated (NH_3_^+^), which prompts the electrostatic interactions with SO_3_^−^ groups of MO, resulting in dye sorption. Considering that all the samples were prepared with the same volume (2.5 mL), the theoretical charge density of the samples is similar, independently of the geometry and presence of hierarchical roughness. However, either the geometry or the presence of hierarchical surface of the hydrogels presented an influence on the sorption kinetics of the model molecule methyl orange (MO) in bi-distilled water.

The effect of geometry was studied by comparing the results of CS-Sph and CS-Cub hydrogels (Figure 4A). The results show that CS-Cub hydrogels demonstrated a faster (1.24 × more rapid) MO sorption than CS-Sph (*p*-value < 0.05). After 6 h of experiments, CS-Cub samples could adsorb ~38% of total dye content. This result indicates that the geometry of each hydrogel influences the dye sorption kinetics. CS-Cub hydrogels possess a larger surface area (1.42 × greater) than CS-Sph, which enhances the contact of dye molecules with the hydrogel, prompting dye removal by diffusion end electrostatic interaction mechanisms explained earlier. Other 3DP systems based on PLA, CS, and/or PVA comprising tablets of different shapes and geometries also demonstrate the relationship between surface area and molecule adsorption [18,24,33]. Their results demonstrate that 3DP parts with greater surface area presented higher molecule adsorption, which agrees with the presented results.

The effect of hierarchical surface can be observed by comparing the results of CS-Sph and CS-Sph_r_ hydrogels (Figure 4A). The results show that CS-Sph hydrogels demonstrated a faster (1.16 × more rapid) MO sorption than CS-Sph_r_. This result indicates that the presence of a hierarchical surface decreased the removal capacity of CS hydrogels, which was not expected. The surface area of CS-Sph_r_ hydrogels was greater (1.88 × greater) than CS-Sph, which it would increase the dye removal capacity as similarly observed by CS samples without hierarchical roughness (CS-Sph and CS-Cub). These alterations might be justified by the differences in charge density (mainly at the surface of CS-Sph_r_ hydrogel) during the neutralization step in strong alkaline solutions. We hypothesized that during the neutralization step, the alkaline solution diffused through the 3DP molds (due to its low infill density of 25% (Figure 1B)) and induced a greater local neutralization, especially in the molds with hierarchical roughness (CS-Sph_r_, in this case). As a result, a lower charge density is observed at the hierarchical rough surface of CS-Sph_r_ when compared to its bulk (Figure 2B). Thus, electrostatic interactions between the polycationic hydrogel and anionic dye are not favored, resulting in lower dye adsorption capacity when compared to hydrogels without hierarchical roughness (CS-Sph and CS-Cub). This hypothesis can be indirectly justified by the visual analysis of the hydrogels after 50 h of contact with MO solutions (Figure 4B). By comparing the hydrogels after dye sorption measurements, it can be observed that the CS-Sph and CS-Cub hydrogels presented a more homogeneous orange color distribution throughout their structure if compared to CS-Sph_r_. In the case of CS-Sph_r_ samples, the structures comprising its hierarchical roughness presented a lighter yellow color compared to its bulk which suggests a different local charge density. This analysis supports the hypothesis mentioned herein. However, further studies must be carried out to confirm this behavior.

Finally, all the samples attained a maximum dye removal capacity of ~75% after one week of contact, and hydrogels were mechanically stable in M0 aqueous solutions for at least one month at room temperature (~23 °C).

### 3.6. Release Profile of the Loaded CS Hydrogels

**Figure 5:**
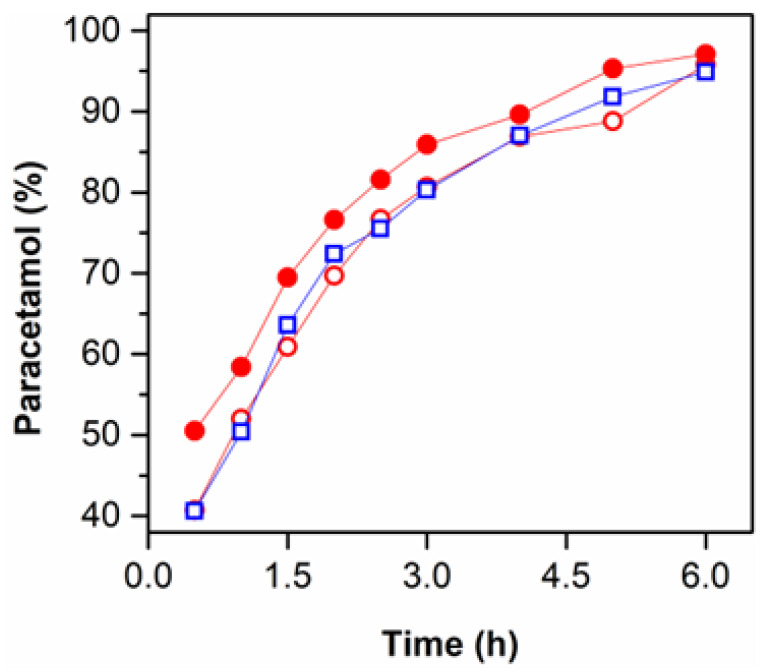
Effect of geometry and hierarchical roughness of CS hydrogels onto the release capacity (in vitro) of non-ionic model molecule (paracetamol [0.03125 mg/mL]) in bi-distilled water at room temperature (~23 °C). The hydrogels were coded as: CS-Sph (○), CS-Sph_r_ (●) and CS-Cub (☐).

The effect of the geometry and surface roughness on the release capacity of indirectly 3DP CS hydrogels was evaluated in vitro, and the results are demonstrated in Figure 5. The average drug loading capacity of all hydrogels was ~8.5 ± 1.4%, independently of geometry and the presence of a hierarchical surface. All the studied samples presented a sustained drug release profile in bi-distilled water. The diffusion mechanism mainly governed the drug release due to the drug concentration gradient. Since paracetamol (acetaminophen) is a non-ionic molecule, electrostatic interactions with the polycationic hydrogels are not favored.

The presence of hierarchical roughness played a significant role rather than the geometry of the samples. In this case, CS-Sph_r_ hydrogels presented a faster (~1.12 × faster) drug release when compared to CS-Sph and CS-Cub samples. These results can be explained due to the differences in surface area for each tested hydrogel. As previously mentioned, CS-Sph_r_ hydrogels possess a larger surface area (up to 1.88 × greater) than CS-Sph and CS-Cub hydrogels. This larger surface area promotes a greater contact between the loaded hydrogel and the aqueous environment. Thus, it induces faster diffusive kinetics of the drug towards the aqueous media, resulting in a “burst release”, mainly at the first time intervals (t ≤ 3 h), which corresponds to ~85% of the drug released. Several authors have demonstrated a correlation between surface area and drug release profile of 3DP materials [17,20,24]. Their results indicate that 3DP materials with higher surface area promote a higher/faster release of different drugs. These observations agree with the results herein presented.

The effect of geometry in the release profile of PMOL was observed by comparing the results from CS-Cub and CS-Sph samples. It is possible to verify that similar results (*p*-value > 0.05) were obtained for both samples, independent of the geometry. Even though CS-Cub samples possess a higher surface area (1.42 × higher) than CS-Sph hydrogels, a dimension threshold might significantly affect release behavior.

Finally, all samples attained 100% of drug release after 24 h of experiments, and no evidence of hydrogel degradation/erosion was observed during the release experiments.

## 4. Conclusions

The present work demonstrated a user-friendly and low-cost process to obtain chitosan hydrogels with different geometries and hierarchical surfaces by indirect 3D printing via FFF technology. This simple approach allows the tuning of physical properties of chitosan hydrogels, such as surface area and the presence/absence of hierarchical roughness. Hydrogels indirectly 3D printed with higher surface areas presented the highest drug release profiles of non-ionic molecules in bi-distilled water. The hierarchical roughness of polycationic hydrogels hindered the anionic dye removal capacity due to local surface charge gradients. These alterations were prompted during the neutralization step, especially in 3DP molds with intricate shapes and hierarchical roughness. Indirect 3D printing can be regarded as an exciting alternative to conventional direct additive manufacturing to explore new polymeric materials that are currently unavailable for the FFF 3D printing approach.

## Data Availability

Not applicable.
